# Adapting and applying student-centered learning in a perfusion clinical rotation

**DOI:** 10.1051/ject/2024001

**Published:** 2024-06-18

**Authors:** James R. Neal, Caitlin Blau, Clint Colby

**Affiliations:** Department of Cardiac Surgery, Perfusion Work Group, Mayo Clinic 1216 2nd St. SW Rochester MN 55902 USA

**Keywords:** Perfusion clinical education, Reflection, Evaluation

## Abstract

While the process of teaching student perfusionists has been in development since the 1950s, the publication of the processes to improve perfusion clinical education has been largely lacking. Publications regarding education from other allied health and medical fields have shown the value of student-centered learning. The use of reflective practice to move perfusion students from thinking about actions after cardiopulmonary bypass (CPB) to reflecting and reacting on actions during CPB is better encouraged by moving from a teacher-centered to a student-centered clinical model. Our institution’s teaching process has developed into a multi-point procedure to make our students into reflective practicing clinicians. Student preceptor evaluations were reversed to allow the students to evaluate themselves first, with feedback from the preceptor given subsequently. Additionally, a biweekly student educational session, where the student chooses a topic and reviews current evidence-based practice, was instituted. The clinical program director serves as the moderator and clinical expert to facilitate problem-based learning during the sessions. Students were also given three skill/experience levels with goals to reach and move through during the rotation. These student levels were also helpful to our preceptors in knowing what each student’s skill level was throughout their rotation. Overall, moving from a teacher-centered to a student-centered clinical rotation has helped make students familiar with reflective practice, self-evaluation, evidence-based practice, and problem-based learning. The incorporation of these processes will hopefully lead students to become lifelong reflective perfusionists.

## Overview

The process of knowledge being passed to students from educators can be classified today into one of two approaches: teacher-centered learning or student-centered learning. The older and more traditional approach is teacher-centered learning. This approach is more focused on lectures, teacher-dictated assignments, and teacher evaluations [1]. Students play a more passive role with this technique. The teacher is the leader and authority in the class, and the students are treated as individualistic and competitive with each other. The emphasis is on the learning of correct answers [1]. Student-centered learning contrasts this approach by placing students in an active role as a partner in learning with the teacher. This is a format pioneered by John Dewey [1]. This includes more multidimensional testing including student self-assessment and ongoing feedback. The emphasis is on the development of a deeper understanding of the subject being studied.

Since the 1950s there has been a change in the education of perfusionists from on-the-job training to a formal university didactic and clinical training model with later incorporation of simulation and animal labs [2]. While there can be much more review and discussion on didactic learning in health care, including perfusion, in this paper we will focus on the clinical education aspect of a perfusionist’s education. Our medical center started taking clinical students in perfusion again, after a 12-year pause, in 2008. In late 2018, our School of Health Sciences, which oversees all clinical rotations in allied health areas, requested that student-centered approaches be considered. It was left to the clinical program directors to move applicable student center approaches forward in their respective programs.

**Figure 1:**
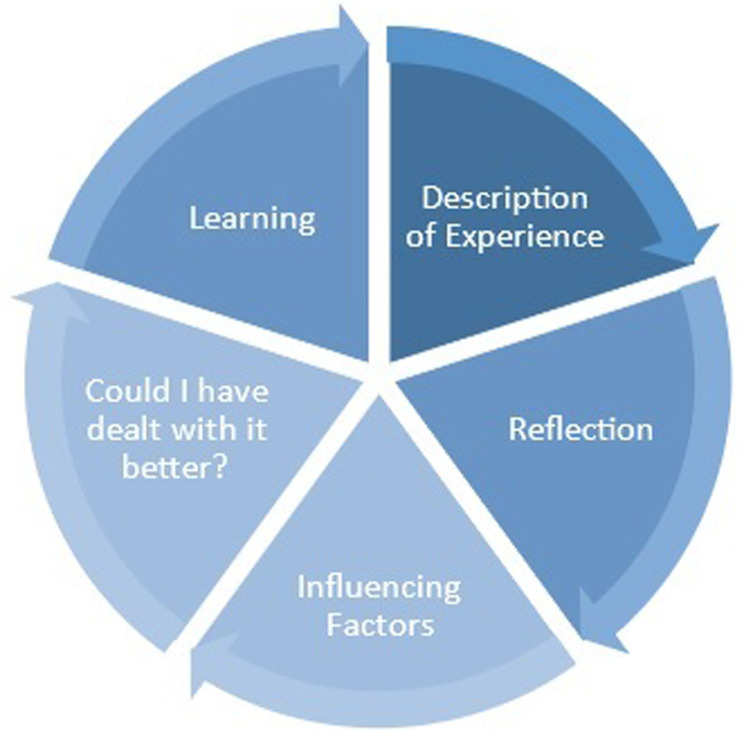
Johns’s model for structured reflection.

**Figure 2:**
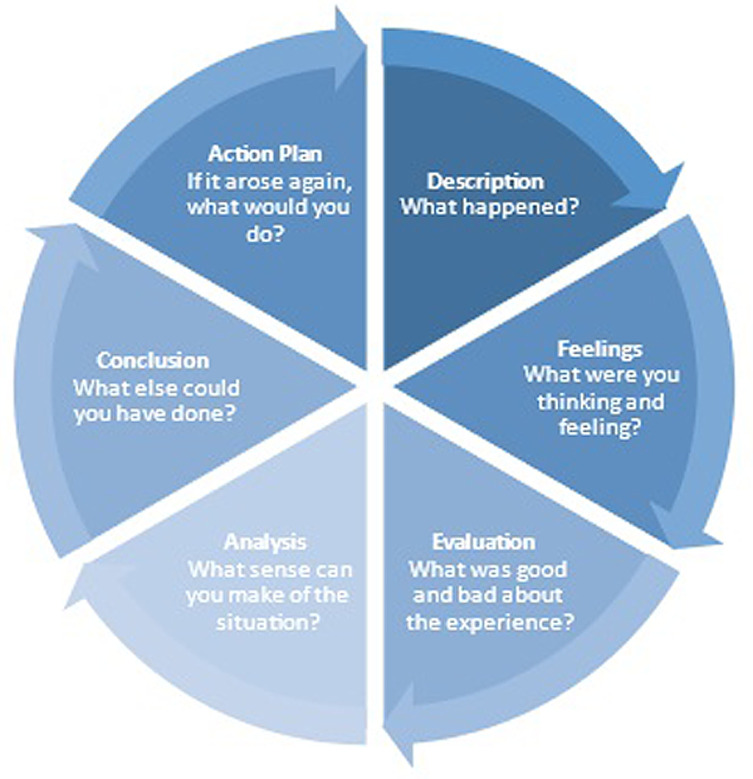
Gibbs’ model of reflection.

In moving to student-centered learning, the student also engages more in self-evaluation and self-reflection. There are many reflective models for students to choose from that have been proposed and studied (Figures 1 and 2). After reviewing many models, the program director felt that John’s Model for Reflective Practice, Gibbs’s Reflective Cycle, and Rolfe’s Framework for Reflective Practice provided the most benefit for clinical perfusion students [3–5]. John’s Model aims to provide a window through which the practitioner can view and focus oneself within the context of their own lived experience. It enables them to confront, understand, and work towards resolving the contradictions within their practice between what is desirable and their current actual practice. In comparison, the Gibbs Cycle moves through a process that starts with describing an event and working throughsix stages to get to an action plan. Rolfe’s framework takes a different approach and encourages practitioners to reflect on their experiences, feelings, and actions, and develop practice accordingly. The goal of these models is to lead students to move from reflecting on action after experience to becoming a clinician who reflecting on action during experience.

Other published articles have shown the benefits of using these processes to improve the abilities of students in the medical field [6, 7]. At our institution the program director provides new incoming students with a resource PowerPoint that describes these three reflective models a month before starting their rotation. Students new to self-assessment and reflection on practice can find this task difficult at first [8]. Reflection should initially be developed in a safe environment where mistakes are tolerated [9].

Although there are no publications outlining how a perfusionist becomes a competent clinician, the ability to evaluate competency in perfusionists has been published [10, 11]. In one of these papers, perfusion intraoperative non-technical skills (PINTS) were used in assessing perfusionists, but not students [10]. Simulated case scenarios used in perfusionist assessment publications, other authors thought, could perhaps be used for student assessment [11]. These authors’ future goals were to develop a minimal standard to reach further pass-through school programs [10]. According to student-centered learning, if this assessment’s goal was to work towards student improvement, then it should incorporate student self-evaluation. Perhaps only at a final test out should it be graded by the school, not the student. Another study’s goal was to utilize what a practicing perfusionist thought of as important clinical skills and apply them to evaluations for students [12].

While performing our literature search, we found another study that looked at perfusion students in simulation. The authors were tracking student eye movements during the simulation. They found an increased tracking of students’ eyes to pO2 after two low pO2 simulations versus two separate different simulations [13]. Another study had perfusion students evaluating their didactic faculty, who were role-playing a perfusion student, with either great or substandard perfusion skills [14]. This has the potential to help students by using a peer review tool. This could also lead students into the process of reviewing and serve as a stepping stone to their own self-evaluation of their cases. These papers rounded out all available literature found on the subjects of perfusionists and perfusion students in the areas of education, evaluation, and assessment.

## Description

Our clinical site currently trains an average of 14 students a year from 3 didactic programs. Depending on their program, these students are at our center for 10–14 weeks. At any given time, we have between 2 and 5 students on-site. By pairing with three programs that have different rotation start dates for students we can maximize volume. Additionally, our daily mix of cardiac cases can then be better assigned based on the ability of the students.

Before students start their rotation with us, the program director sends out our site’s perfusion clinical rotation orientation program book to the students. Additionally, they send them a site-specific document called the body of knowledge sheet. In our years of experience, we have found that once students begin their clinical, they occasionally forget important concepts that they learned earlier in the didactic portion of their program. We give them this document with educational topics to review including drugs, tubing volumes per foot, cardioplegia types, and unique cardiac surgical procedures that we perform at our institution that may not have been covered in school. Less review is needed by the preceptors and director since providing the body of knowledge sheet. This is mentioned based on observation and consensus only, however the change was very noticeable.

The clinical program director will assign students to preceptors. Ideally, the assignments are for a week at a time. These assignments are made after looking at who is available based on position in preceptor rotation and vacation time off. All students spend one week each with the program director as a preceptor. In most instances, each student spends one week with a preceptor but no more than two weeks with the same preceptor. This allows the students the ability to see and work with as many preceptors as possible. We currently have 24 perfusion preceptors at our center.

Another area that is covered during rotation at our institution is the ability to give students time to lead a biweekly education hour in which two topics are chosen. These are typically focused on special cases such as sickle cell anemia, cold agglutinins, pregnancy, and others. Students select topics, find articles that promote evidence-based practice (EBP), and review the most important parts of topics for perfusionists to think about or plan out how to deal with the topic. EBP for practicing perfusionists has been reported on in a low sample size study. It showed that higher education level and work status at an academic center increased EBP [15]. The goal of these sessions is to promote problem-based learning (PBL) and have the students construct mental models of the perfusion world. Students lead the discussion with questions and are aided by the program director to provide PBL scaffolding to make some support and connections for the learners to further their own ideas. The program director also prompts the students to think about what the most critical areas are that a perfusionist should think of for these topics. The students then take the lead in answering and incorporating their own thoughts. The program director then offers clinical input on the topic. The goal of these interactions is for students to develop skills and the ability to look for quality articles that would be viewed as the best articles for developing EBP guidance for a perfusionist. We also ask that the students not refer to our institutional guidelines or look at the references in those guidelines, as that amounts to getting the cliff notes on the topic and defeating the goal of the education hour. Over the course of having students rotate the topics are usually repeated, but not with the same students present.

By partnering with multiple programs, the starting dates of students are staggered. This allows for previous students who have already started to obtain experience before we receive more students. This allows the new students to participate in less intense and more straightforward cases, thus giving the experienced students the more difficult, challenging cases that they have attained the ability to perform. Similar processes have been used successfully with nursing students [16]. The best analogy for this is using an escalator as a model. Students enter at the bottom of the escalator and work their way up to the higher level. This allows more students to get on at the bottom. The escalator does not stop, but as students leave the escalator (rotation) it is filled with more students. The mid-level students taking over as senior students (top of escalator) that have the most experience are able to assist the newer students and provide helpful tips.

With a large number of preceptors and rotating students at our institution, utilizing a level system was identified as a way to provide students goals to reach and preceptors an idea of the student’s aptitude. There are three levels, labeled numerically: 1, 2, and 3. All students start at level 1 with the goal of reaching level 3 by the end of their rotation. Ideally, students in level 1 are given more straightforward cases like coronary artery bypass grafting and valve-only cases. As students move up levels the cases get more complex. Level 2 cases may include redo sternotomy and complex valve cases. Level 3 cases are the most difficult and may involve deep hypothermic circulatory arrest with retrograde or antegrade cerebral perfusion (Table 1). The amount of assistance from the preceptor is expected to decrease as student levels increase. Assessment for promotion up the levels is done by the program director with feedback from the students and their recent preceptors.

**Table 1:** Student level table.

Level 1 Student
Entry Level for all students
They are in the first week up to around week 3–5. Learning setup and priming (need assistance from preceptor on this task perhaps). Still needing to complete observations of some surgeons and could do this as secondary cases (might need to forgo clean up on the first case to accomplish this). EPIC interaction is limited to simple tasks of basic buttons and documentation of items with help from preceptor (EPIC should be the last item to have a student at this level focus on). Assigned to normally straightforward CABGs, valve, cases over this level should be considered observational, or a case that will need a large amount of preceptor interaction. Assistance from the preceptor in setup and tear down in the OR is needed. If here for pediatric cases they can start those after week 2.
Level 2 Students
This would be in the range of a week 4/6 to week 8/10 student. Completed observations with all surgeons. Full ability to setup and prime effectively (may have a 630 start time instead of 600 start time at this point on Monday 700 start time). If here for pediatric cases then should be getting into the halfway point of pediatric cases needed. EPIC interactions can increase to charting most of the timers, notifications, cardioplegia, and drug administration on CPB and I/O area after CPB. Assigned to straightforward and move difficult cases including LVAD insertions, redo cases with multiple procedures needed (this may include hemi arch cases with a fair amount of assistance from the preceptor). Some assistance with regard to OR setup and teardown.
Level 3 Students
This would be in the range of students over week 7–11 (depending on pervious number of rotations). Full ability to setup with a 630 start time 700 on Mondays. Finishing pediatric cases/observations. Normally assigned two weeks of ICU time with ECMO and VAD patients. Given a choice week to pick cases and work with the perfusionist assigned to that case (if certain cases or preceptors are desired then discuss with the Program Director and charge perfusionist). Minimal or no assistance with regard to OR setup. EPIC use during the case can be done with limited assistance from the preceptor (perhaps some help with medication documentation) (making sure that prime, checklists, staff, and billing are always the preceptor’s responsibility). Cases may include most all cases and may include the most complex cases (*i.e.*, total arch cases, TAA cases, and may include high-risk patients (depending on the students’ previous amount of experience). Early rotation students might not make it to this level of case ability, but students close to graduation at Level 3 should be able to do complex cases with limited assistance from the preceptor.

In order to make evaluations more student-centered instead of preceptors filling out the evaluation the student takes the lead on this endeavor. This is not to lessen the preceptor’s responsibilities but rather designed to make the evaluation and the learning points “stick” for the student. The student completes their full required didactic programs evaluation including the grading areas for individual tasks. The student then adds in at least three areas where they learned something new, did a task well, or need to improve on a task. Each of these areas is required to be expanded upon with a minimum of three sentences. The goal is that the subject will be identified, framed, and a conclusion formed within these sentences. By doing this the student has completed a mini-reflection three times. At this point, the preceptor reviews the whole evaluation with the student. If additions or changes in grading areas are needed the preceptor will discuss this with the student and the student will make the changes. These evaluations are also done immediately following the case, usually while waiting for the bypass circuit tubing to be handed back. This is done at least 30 min after the arterial cannula is removed and after chest closure. If the preceptor is relieved during the case, then the relieving preceptor is responsible for completing the evaluation with the student. The preceptor being relieved will give a report on how the student has been doing on the case verbally or via a paper copy of the student’s evaluation. In any case, timely feedback and completion of the student’s evaluation are critical for their improvement and information retention.

Clinical student learning would not be possible without preceptors. For preceptors to be as effective as possible some baseline education and common practice benefit both the student and preceptor. At our institution, the program director makes a yearly education module to touch on an educational approach or theory for the preceptors to review. These include a podcast and additional website material from the medical center’s school requiring about 1 h of the preceptor’s time. Incorporating and adapting our medical center’s established content to meet our needs prevents us from having to invent our own content, thus saving resources while aligning with our institution’s medical center’s school. New preceptors at our institution also complete all previous years’ training. We currently have five modules which include general handbook policy, self-reflection, making learning stick, learning preferences, and others. Previous studies in perfusion and allied health preceptor training have similar approaches with one study having 50 h of training and another study with five modules to complete [17, 18]. An additional study showed the importance of giving nursing faculty the background and helpful implementation techniques of student-centered learning [19]. Another study had students give feedback on preceptors, which is something that we have not done at our institution [20]. Students do have an exit interview that is completed with the student and perfusion clinical program director.

## Discussion

With student self-evaluation, preceptors get to see how students rate their skills and can coach those who too self-critical. Preceptors can also guide those who are overconfident before graduating and becoming a practicing perfusionist. At our center, it is common for students during the first rotation to grade themselves lower than the preceptor does. Some students take a little time to acclimate to grading themselves. As students get closer to final rotations the areas of improvement become more detailed and focus on promoting the essence of a competent perfusionist.

Our goal of making improvements to the parameters and techniques that we use and offer to the students is to ultimately get them into a position where they will become lifelong reflective, evidence-based practicing clinicians. During the process of mastering the abilities to become a competent perfusionist, many experienced perfusionists used some of these techniques without fully understanding their names and the evidence behind them. By having a more formal process, it becomes a more easily reproducible and transferable process to help future perfusionists maximize their abilities to improve themselves and care for their patients.

While we do not have any publishable data showing this improvement since starting these student-centered practices, anecdotally the performance of students has improved over the course of their rotations more than what we have seen before these changes were implemented. Since instituting the use of reflection and self-evaluation 4 years ago, we feel it has reduced the number of repetitive student issues during cases. Certainly, the ability to gather data would be something that could be done in the future to be able to tease out the differences in perfusion student learning. At this time it was seen as a delay and an obstacle to getting the general information out to the perfusion community at large. This would be a limitation of this paper.

Many of these changes that were made are minor to the impact of perfusion groups but have the potential to lead to major improvements to the ability of preceptors to better impact the knowledge growth of students rotating at their institution. One of the goals that we had was to make students turn into new graduate perfusionists with the ability to reflect and evaluate at the moment when patients need care instead of evaluating and reflecting after the case has been completed.

In a large academic center, the ability of all preceptors to be updated on a particular student’s abilities is limited. By creating a simple system to identify the current ability of a student, preceptors have a better understanding of the student’s abilities. Three levels were selected with the belief that preceptors would be likely to remember the differences between levels and where a student is within the level system. This is compared to an overall more complex five to ten-level approach where preceptors would lose the ability to remember the major differences between levels. Another benefit of the level system is that it gives additional goals and milestones for the students to target and reach. Overall response from students over the years has been positive to student levels and they have genuinely been excited as they move up and change levels. While a smaller institution might have fewer students and individual preceptors working more frequently with a specific student. The ability to have additional targets and goals for students to reach would still make this technique applicable for even smaller institutions to try.

Providing the students with information and requesting them to perform reflective practices and self-evaluation on their own time takes a certain amount of trust from the preceptors and program director that the students will participate. Currently, we do not keep track or have the students record this time, although with discussions of level increases it is often referenced to ask how things are going with these reflections and evaluations. Certainly, a more formal process in this area could be designed if an institution desired or didactic program required this. Previous studies, in nursing, show students do adapt to student-centered learning and test scores improved with this approach [21].

Once a student graduates and starts independently practicing, the ability for realistic grading of their performance by others diminishes. If a student never developed the ability to self-evaluate their performance, it is a skill that would be more difficult to implement at this point. By allowing students the ability to grade themselves first before the preceptor the preceptor can then see what the student is thinking about their progress. The preceptor can then offer more meaningful insights on where the student’s practice needs to improve. This could prevent students from being too critical of their performance or from being overly confident of their abilities.

Having incorporated student-centered learning into our practice including areas of EBP, PBL, self-evaluation, and self-reflection we feel that this has offered the students a more meaningful rotation and given them the tools to become lifelong reflecting and evaluating perfusionists that use EBP. Hopefully, some of these students with experience of their own will now be able to teach new students and continue this line of processing to future perfusion students for the betterment of patient care [22].

## Data Availability

No data was analyzed in this paper. Given this, we do not have a repository of data for supplementary material to acknowledge.
